# Character of woodland fragments affects distribution of myriapod assemblages in agricultural landscape

**DOI:** 10.3897/zookeys.930.48586

**Published:** 2020-04-28

**Authors:** Ondřej Horňák, Andrej Mock, Bořivoj Šarapatka, Ivan Hadrián Tuf

**Affiliations:** 1 Palacký University Olomouc, Faculty of Science, Department of Ecology and Environmental Sciences, Šlechtitelů 27, 78371, Olomouc, Czech Republic Palacký University Olomouc Czech Republic; 2 Pavol Jozef Šafárik University, Faculty of Science, Institute of Biology and Ecology, Šrobárova 2, 04154, Košice, Slovakia Pavol Jozef Šafárik University Košice Slovakia

**Keywords:** activity-density, area, Chilopoda, Diplopoda, landscape elements, species richness

## Abstract

Fragments of woodland fulfil many irreplaceable functions in the agricultural landscape including being the main source of biodiversity of soil invertebrates. Due to intensive farming and land use changes, especially in the second half of the 20^th^ century, fragments of woodland in agricultural landscape almost disappeared. This has led to a decrease in the diversity of invertebrates, especially those for which the presence of these woodland habitats in the landscape is a key element for survival. The aim of this study was to evaluate the importance of fragments of woodland (characterised by their area, vegetation structure, the amount of leaf litter layer and soil moisture) on the distribution of centipedes and millipedes (Myriapoda) in the agricultural landscape of South Moravia (Czech Republic). Myriapods were collected using pitfall traps during summer in 2016 and 2017. Results showed that activity-density of myriapods is positively correlated with thickness of the leaf litter layer. Moreover, the species richness of centipedes is positively correlated with increasing size of fragments of woodland although higher centipedes’ activity-density was found in rather uniform woodlands in term of diversity of tree species.

## Introduction

Hedgerows, wood fragments, windbreaks, and other wood elements represent an integral part of the agricultural landscape, defined as a mosaic of fields and uncultivated natural or semi-natural areas (Burel 1996). They were created by human planting primarily or they are remnants of formerly wooded landscape (Burel and Baudry 1989). The species composition of flora and fauna of these forest elements differs based on historical rural development and agricultural practices (Burel 1996). In the agricultural landscape, these elements fulfil many important functions. In addition to their importance in protecting against water and wind erosion (Burel and Baudry 1989; Baudry et al. 2000; Stašiov et al. 2017), they also represent a major source of invertebrates’ biodiversity in the landscape (Diekötter et al. 2007).

The presence of invertebrates is very important as they provide many irreplaceable ecosystem services. One of the major groups of ground dwelling invertebrates in this regard are myriapods (Myriapoda). Millipedes (Diplopoda) are in most cases detritovores, saprophages or phytophages (Kime and Golovatch 2000). They consume decaying plant residues that break into smaller particles, thereby mediating this dead material to microbial decomposition (David and Handa 2010; Riutta et al. 2012; David 2014; Bogyó et al. 2015; De Smedt et al. 2016, 2017, 2018). Centipedes (Chilopoda) are soil predators that feed on larvae and adults of other small invertebrates and thus significantly reduce the abundance of other invertebrates (Voigtländer 2011). Both these groups of myriapods are also sources of food for other animals (Stašiov et al. 2017).

For ground dwelling invertebrates, that use uncultivated areas, hedgerows and fragments of woodland are indispensable as they provide suitable habitats for the survival of their populations in the agricultural landscape (Agger and Brandt 1988). They are especially important for species that are strictly bound to these habitats, but also for species that spend a certain part of their life cycle on them (Burel and Baudry 1990, 2005). Higher heterogeneity, undisturbed habitats and the presence of vegetation cover also affects the availability of food sources for invertebrates (Previati et al. 2007).

Fragments of woodland and hedgerows were for centuries under the influence of transformations by farmers, who often evaluated them as being worthless and thus they eliminated them (Burel and Baudry 1989). The main change in European landscapes occurred in the second half of the 20^th^ century (50s–80s) (Burel and Baudry 1990), particularly in terms of the structure of agricultural landscapes (Weibull et al. 2003). In countries in which agriculture collectivization was applied (e.g., former Czechoslovakia) such changes were even more dramatic (Šarapatka and Štěrba 1998; Havlíček et al. 2018). Under the influence of specialization and intensification, there was simplification of the landscape and redistribution of land, where emphasis was placed on the extension of arable land. This management led to a change in the structure of landscape (fragmentation, decrease and uneven distribution of hedgerows and fragments of woodland) and habitat quality, which was reflected also in local species richness (Agger and Brandt 1988; Wade et al. 2003; Dauber et al. 2005; Previati et al. 2007).

Such long-term landscape management caused many negative effects, which also manifested themselves in the ground dwelling invertebrates. Previati et al. (2007) point to the increased impact of disturbances associated with intensive field cultivation that have been recognized to reduce the species richness of invertebrates. Moreover, loss of habitats, their fragmentation or reduction of size lead to the species extinction or increased susceptibility to their disappearance (Fahrig 2003; Ewers and Didham 2005; Honnay et al. 2005).

Quality natural resources and ecosystem services that directly provide fragments of woodland and hedgerows or invertebrates, which are supported through presence of these habitats, are indispensable for agriculture. Their rational use and proper management in the landscape should therefore be given special attention (Meeus 1993; De Smedt et al. 2017). Based on this, we tried to evaluate the relationship between fragments of woodland (their size, vegetation characteristics, thickness of leaf litter and moisture) and the activity-density and species richness of myriapods.

## Materials and methods

### Description of study sites

Research was realized in agricultural landscape of Southern Moravia in the vicinity of the villages of Šardice (48°58'N, 17°2'E), Stavěšice (49°0'N, 17°2'E), Čejč (48°57'N, 16°58'E), and Hovorany (48°57'N, 17°0'E). The studied sites consisted of 38 pre-selected isolated wood fragments and hedgerows. The surrounding matrix of these habitats consisted mainly of arable land, vineyards and partly permanent or temporary grasslands.

Wood fragments represented the remains of lowland broadleaf forests, coastal tree vegetation around the streams, or artificially planted orchards, or wood linear elements forming the natural boundaries between lands. Many patches were largely invaded by self-seeding black locust (*Robinia pseudacacia*), blackthorn (*Prunus spinosa*) and elderberry (*Sambucus nigra*). Remains of large wood stands were mostly made up of linden (*Tilia* spp.), birch (*Betula pendula*), ash (*Fraxinus excelsior*), pine (*Pinus silvestris*), chestnut (*Aesculus hippocastanum*), oak (*Quercus* spp.) or maple (*Acer* spp.). Other hedgerows were often made of walnut (*Juglans regia*), cherry (*Prunus* spp.) or poplar (*Populus* spp.).

Five pitfall traps were placed at each site, these consisting of a plastic cup of a volume of 3 decilitres (diameter 7 cm, high 13 cm) buried uniformly with the soil surface. The traps were half filled with 4% formaldehyde as a fixative solution and were covered by metal sheets. The pitfall traps were arranged in line spacing 10 m inside a wood patch. Some linear wood strips were only ca 10 m wide; in this situation a line of traps passing through the middle of the strip. In larger patches, the line was placed at least 10 m far from the edge. Traps were installed on the sites for three weeks during June to August of 2016 or 2017. Caught centipedes and millipedes were identified to species level.

### Environmental variables

The distribution of invertebrates in the landscape was assessed in relation to several selected environmental variables:

(1) *size of the sites*, which ranged from 0.04 to 7 ha (measured using Google Earth software),

(2) *density of the canopy of trees*, evaluated at scale 1–4, when level 1 is for canopies covering sky from 0–25%, level 2 for sky covered by canopies at level 26–50%, level 3 for covering sky at level 51–75% and level 4 for canopy covering sky by 76–100%, covering was mean of estimations by naked eyes of three independent persons,

(3) *the percentage coverage of surface by herb layer*, estimated as mean of three independent estimations (rounded to tens of percent),

(4) *dominance of grasses in herb layer*, evaluated by the same methods as previous parameter,

(5) *thickness of leaf litter layer*, measured in centimetres as the mean of three measures on different points at each site,

(6) *soil moisture*, measured gravimetrically during installation of traps,

(7) *species richness of trees*, expressed as number of tree species creating evaluated wood fragment and

(8) *black locust dominance* in tree layer, estimated as the mean of three independent estimations with 10% accuracy.

### Data analysis

Constrained ordination and canonical correspondence analysis (CCA) were used to analyse the activity-density and species richness of the invertebrates (dependent variables) in relation to individual environmental factors (independent variables). To assess the trends of the ordination diagrams, we used a generalized additive model (GAM). All analyses were performed using the CANOCO 5 program (Šmilauer and Lepš 2014).

## Results

Altogether 245 individuals of centipedes (Chilopoda) in 11 species were caught, and 304 individuals of millipedes (Diplopoda) in 7 species (Tab. 1). The mean catch is 0.4 centipede and 0.5 millipede per trap per week, respectively. The most dominant centipede species, *Lithobius forficatus* and *Lithobius microps*, represented together 85% of caught individuals. Among the millipedes, *Polydesmus complanatus* showed the highest activity (96% of all sampled millipedes). Number of species of trapped centipedes per site usually reached values of 1 or 2 with minimum 0 (three localities) and maximum 5 (once). Due to dominance of *P. complanatus*, this millipede was usually the only species caught and 14 sites were without surface-dwelling millipede activity during our research.

**Table 1.** List of the centipedes and millipedes caught on 38 studied localities, total number of individuals caught by 5 traps during 3 weeks and number of localities, at which species was recorded.

Taxon	Individuals	Localities
** Chilopoda **	**245**	**37**
*Geophilus electricus* (Linnaeus, 1758)	3	3
*Lamyctes emarginatus* Newport, 1844	3	3
*Lithobius aeruginosus* L.Koch, 1862	2	2
*Lithobius austriacus* Verhoeff, 1937	1	1
*Lithobius crassipes* L.Koch, 1862	2	2
*Lithobius cyrtopus* Latzel,1880	1	1
*Lithobius erythrocephalus* C.L.Koch, 1847	8	6
*Lithobius forficatus* Linnaeus, 1758	164	32
*Lithobius micropodus* (Matic, 1980)	2	2
*Lithobius microps* Meinert, 1868	45	18
*Lithobius mutabilis* L.Koch, 1862	14	9
** Diplopoda **	**304**	**24**
*Blaniulus guttulatus* (Fabricius, 1798)	1	1
*Brachyiulus bagnalli* (Curtis, 1845)	1	1
*Brachyiulus lusitanus* Verhoeff, 1898	1	1
*Cylindroiulus boleti* (C.L. Koch, 1847)	1	1
*Enantiulus nanus* (Latzel, 1884)	1	1
Polydesmus cf. denticulatus C.L. Koch, 1847	6	3
*Polydesmus complanatus* (Linnaeus, 1761)	293	22

Using canonical correspondence analysis (CCA) and subsequent generalized additive models (GAM), we tested the species richness and activity-density of both myriapods in total, and centipedes and millipedes independently in relation to individual measured environmental characteristics.

**Figure 1.**
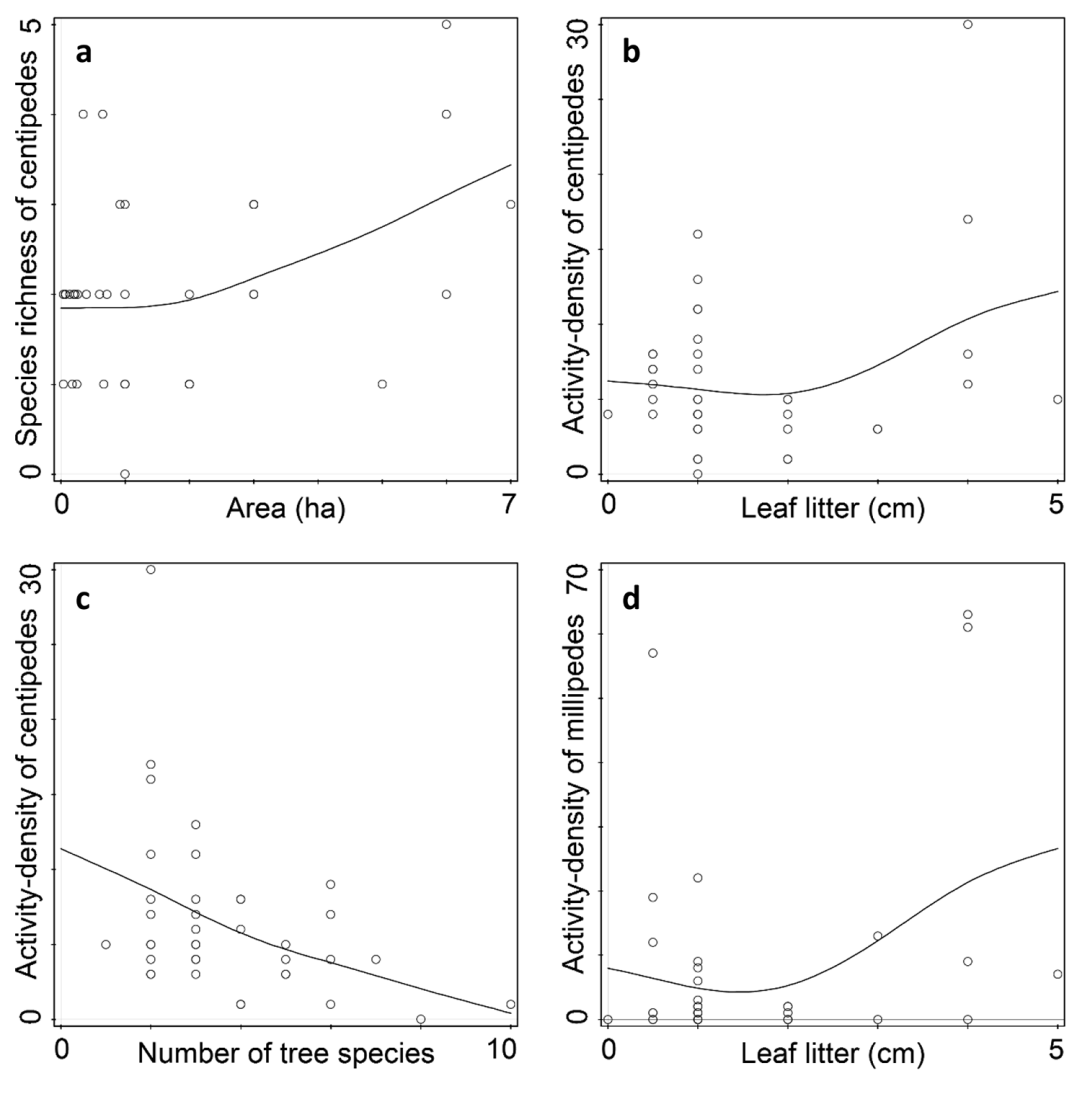
GAM plots evaluating effect of selected measured environmental factors to communities of centipedes and millipedes **a** relationship between size of wood fragments and number of centipedes’ species trapped **b** relationship between thickness of leaf litter and number of centipedes trapped **c** relationship between species richness of trees in sites and number of centipedes trapped **d** relationship between thickness of leaf litter and number of millipedes trapped. Circles on diagram represent individual fragments of woodland.

In independent evaluation of centipedes’ distribution, the measured environmental variables explained 22.2% of the pattern of distribution. From the tested environmental characteristics, the size of fragments of woodland (F = 3.8, p = 0.031), the thickness of the leaf litter (F = 3.4, p = 0.045) and species richness of the tree floor (F = 3.6, p = 0.038) proved to be significant (Tab. 2). The number of recorded species of centipedes increased with the increasing size of the fragments of woodland (Fig. 1a), although their activity-density was not affected (p > 0.05). Activity-density but not species richness of centipedes was positively correlated with increasing leaf litter thickness (Fig. 1b). On the other hand, a significant decrease in activity-density was observed in localities with higher species richness of the tree floor (Fig. 1c). There was also a decline in species richness of centipedes, but the statistical model in this case came out slightly above the significance level (F = 2.9, p = 0.07).

**Table 2.** The effect of measured environmental factors to myriapod communities. Effects to its activity-density and species richness for both taxa are presented independently.

	Chilopoda				Diplopoda			
	Activity-density		Species richness		Activity-density		Species richness	
	F	p	F	p	F	p	F	p
*area of wood fragment*	1.60	0.208	3.80	0.031	1.40	0.257	2.40	0.104
*canopy coverage*	0.07	0.931	0.45	0.643	2.40	0.105	2.50	0.101
*herb layer coverage*	1.40	0.257	0.60	0.556	0.38	0.687	1.50	0.248
*grasses dominance*	2.10	0.144	1.50	0.237	1.50	0.229	0.69	0.506
*leaf litter thickness*	3.40	0.045	1.10	0.336	4.20	0.023	0.56	0.576
*soil moisture*	0.91	0.588	2.30	0.113	0.44	0.650	0.42	0.659
*tree diversity*	3.60	0.038	2.90	0.071	1.10	0.341	1.20	0.326
*black locust dominance*	0.63	0.538	1.10	0.343	2.50	0.095	0.62	0.543

In independent analysis of millipedes’ distribution, the measured variables explained 23.5% of variability in their distribution pattern. A significant response of millipedes was to leaf litter thickness (F = 4.2, p = 0.023) in terms of their activity-density, i.e., with increasing thickness of leaf litter the number of captured individuals increased (Fig. 1d). Analysis of species response curves showed that effect of leaf litter was reflected only in *P. complanatus* (F = 4.4, p = 0.019). No other factor significantly affected millipede activity-density (Tab. 2). In addition, the species richness of millipedes was not affected by any factor.

## Discussion

We studied the distribution of millipedes and centipedes inhabiting fragments of woodland and hedgerows in intensively used agricultural landscape. We focused on several characteristics of the environment, which, as we supposed, could affect the species richness and abundance of these myriapods. Leaf litter thickness, area size and species richness of tree species affected myriapod assemblages.

Although number of trapped individuals seems to be rather low, very similar number of trapped centipedes (0.45 per trap per week) and millipedes (0.52 respectively) were recorded in hedgerows in Slovakia (Stašiov et al. 2017) too. Moreover, the design of study, i.e., methods (the solely pitfall trapping) and term of study (dry summer months), was chosen with respect to more groups of soil macrofauna (spiders, ground beetles, woodlice) and was not aimed to describe myriapod communities in all details (Tuf 2015). It is possible there are other species of millipedes and centipedes living in the soil and less tolerant to desiccation, but such isolated sites are able to colonize surface active species more easily (De Smedt et al. 2018). High activity and dominance of *P. complanatus* among millipedes is not surprising. This species is one of the largest millipedes among Central European species and is more resistant to desiccation. It can stay active during summer months (when research was done) (De Smedt et al. 2017).

### Leaf litter

Myriapods usually occur in the leaf litter layer, typically in deciduous forests and benefit from its greater thickness and soil surface coverage. For millipedes, leaf litter is a direct source of food, while centipedes are affected indirectly through the availability of prey (Voigtländer 2011; Riutta et al. 2012; Bogyó et al. 2015). Moreover, predatory centipedes are mostly generalist, not dependent on the availability of a specific diet (Blackburn et al. 2002; Scheu et al. 2003) and thicker leaf litter layer can provide them a wider range of prey (Voigtländer 2011). This was also confirmed in our case, in which the thickness of leaf litter was correlated positively with activity-density of both millipedes and centipedes. This conclusion is also supported by Gava (2004), reporting that myriapods were more densely located in the lower layers of the litter, which are more favourable because of more stable microclimatic conditions. The litter layer is most important during the dry season, and this research was done in dry summer months.

### Diversity of trees

Millipedes are influenced by the quality of leaf litter consumed, while higher tree species diversity often contributes to greater variability in food supply (Stašiov et al. 2012, 2017). However, we have not observed any positive effect of tree species diversity on the improvement of activity-density or species richness of millipedes. On the contrary, with increasing number of tree species, activity-density of millipedes had a tendency to decrease, although this relationship was not significant. Despite the higher diversity of trees, in the habitats may dominate tree species, which are not preferred as a source of food by millipedes. Therefore, the expected positive effect of higher species richness of tree species may be limited (Stašiov et al. 2017). On the other hand, when almost all millipedes belonged to one species, the effect on diversity cannot be correctly tested. It is also necessary to point out that those artificial fragments were usually of highest tree diversity (e.g., abandoned gardens with diverse fruit trees) and more nature-similar woods were forested by few tree species.

Centipedes showed that with increasing richness of tree species their activity-density decreased. At the same time, in habitats with a lower number (2–4) of tree species black locust dominated. Similarly, Štrobl et al. (2019) noted a positive effect of black locust on some invertebrates, especially the species of open habitats, which benefited from the specific microclimatic conditions of these stands. Moreover, black locust, due to its ability to fix atmospheric nitrogen, has an increased content of nitrogen and other nutrients in leaf litter (Rahmonov 2009), although Berthold et al. (2009) state it may be more difficult to degrade. Thus, the rate of decomposition between acacia leaves and leaves of other tree species may not be entirely comparable. Despite this, an increase in food supply for predatory centipedes may result, as e.g., Stašiov et al. (2012) noted higher species richness and equability of millipedes with increasing nitrogen content, and they also mention the positive effect of leaf litter quality on the abundance of these decomposers. Also, in our case, the activity-density of millipedes correlated positively with black locust, although this dependence was not significant. On the other hand, the long-term effect of black locust leads to acidification and decrease of nutrients in soil (Berthold et al. 2009) and, as reported by Scheu and Poser (1996), with increasing acidity the density of soil macrofauna generally decreases. However, this negative effect of black locust can be mitigated through a higher nitrogen content in the litter, which improves the quality of food for decomposers and supports microbial activity (Vasconcelos and Laurance 2005; Stašiov et al. 2012; De Smedt et al. 2018). Scheu and Poser (1996) also mention an increase in the density of the springtails (Collembola) with higher soil acidity, which may increase the food supply for centipedes.

### Size of area

The increase in species richness with area size is mostly attributed to increasing environmental heterogeneity (Tews et al. 2004; Báldi 2008). However, in many cases, the species richness of individual taxonomic groups can be influenced by their different life strategies or specific habitat factors (Báldi 2008). In our case, the influence of the area size was recorded only in terms of species richness of centipedes, which positively correlated with increasing area size. One explanation could be the effect of specific microclimatic conditions of fragments of woodland in the agricultural landscape. Since humidity and temperature are the main limiting factor for centipedes (Voigtländer 2011, Kicaj and Qirjo 2014), the more favourable and more stable conditions provided from this point of view, were rather larger areas, which were typically more forested with greater shading. Smaller areas often had a more open character and due to their size and shape, acted as a drier forest edge habitat throughout their area (De Smedt et al. 2017).

Accordingly, Young and Mitchell (1994) state that as the habitat decreases, the area acting as the inner part of the forest (more stable temperature and light conditions) also decreases and the edge effect becomes more significant. The habitat smaller than 1 hectare then acts as a marginal habitat. As well, Riutta et al. (2012) mention the differences in microclimatic conditions between the edge and the interior of the fragments, where evapotranspiration grows at the edges and thus reduces the moisture of the soil and leaf litter. On the other hand, forest edges host the most abundant (or active) communities of millipedes (Bogyó et al. 2015; De Smedt et al. 2017) contrary to forest interiors. Due to these two factors affecting communities of myriapods in contradiction, finding a simple effect of forest site area on myriapods is not easy.

### Other habitat characteristics

None of the other environmental characteristics investigated has been shown to be significant, although, for example, moisture is one of the major factors affecting the distribution of myriapods, as reported by many authors (Gava 2004; Tajovský and Wytwer 2009; Wytwer et al. 2009; Voigtländer 2011; Stašiov et al. 2017).

However, there were no substantial differences in moisture within the sites (moisture of soil ranged from 10 to 41% as measured during installation of traps), which may be one of the reasons why moisture did not significantly affect myriapod distribution (Riutta et al. 2012). Similarly, Lazorík and Kula (2015) also did not confirm the significant effect of moisture on centipede and millipede assemblages. Myriapod assemblages can also be influenced by other characteristics such as temperature (Voigtländer 2011), pH or organic carbon and nitrogen content in the soil (Scheu and Poser 1996; Stašiov et al. 2012, 2017). According to Grgič and Kos (2005), the heterogeneity of the internal horizontal structure of the stand is a key factor that also affects centipede diversity. Nevertheless, these characteristics were not the subject of our research. Finally, we must also consider the effects of the surrounding intensively used agricultural landscape that can specifically reflect on the characteristics of fragments of woodland and hedgerows (Paoletti et al. 2010).

## Conclusions

The results of our study show that the leaf litter layer may be one of the important characteristics affecting the surface dwelling myriapod assemblages inhabiting the fragments of woodland in the agricultural landscape. In addition, the size of individual fragments of woodland and the species richness of the tree canopy also have a noticeable effect. These habitat characteristics work together with many other factors, and the interpretation of particular conclusions is often difficult. However, we believe that specific environmental conditions of fragments of woodland are a key element for distribution of myriapod assemblages in intensively cultivated agricultural landscape.
